# Nonsurgical Management of an Extensive Endodontic Periapical Lesion: A Case Report

**DOI:** 10.22037/iej.2017.24

**Published:** 2017

**Authors:** Amirabbas Moshari, Mehdi Vatanpour, Ehsan EsnaAshari, Mehrsa Zakershahrak, Afsoon Jalali Ara

**Affiliations:** a * Department of Endodontics, Dental Branch, Islamic Azad University, Tehran, Iran; *; b * Dentist, Tehran, Iran*

**Keywords:** Cyst, Endodontic Therapy, Nonsurgical, Periapical lesion

## Abstract

Long-term success of endodontic treatment is dependent on adequate and appropriate cleaning and shaping of the root canal along with proper and correct obturation of the entire prepared space. This article aims to report an exceptional non-surgical and orthograde endodontic treatment of maxillary right central incisor with an extensive radiolucent lesion in a 17-year-old male. Six and 20-month follow-ups showed significant changes, including bone formation and periapical healing within the lesion. The patient was asymptomatic. After 20 months, complete radiographic and clinical healing of the periapical lesion was observed.

## Introduction

Pulp disease and bacterial infection of the pulp space, result in periapical lesions [1]. These lesions are usually found during routine radiographic examinations or followed by patient’s extreme pain sensation [2]. Periapical lesions are mostly classified as radicular cysts, dental granulomas or abscesses [3, 4]. Among all periapical lesions, the incidence of cysts varies from 6% to 55% [5]. Also, the occurrence of granulomas spans from 9.3% to 87.1%, and of abscesses from 28.7% to 70.07% [6]. According to clinical evidence, lesions that are larger in size, are most likely radicular cysts. Still, some of these large lesions may appear to be granulomas [7] .

The preliminary purpose of all endodontic procedures, specially cleaning and shaping, is to eliminate necrotic tissue and infective bacteria [8]. Root canals are usually not adequately prepared in apical third [9] and thorough disinfection of this zone cannot be expected [10-13]; therefore, complete obturation of the prepared, cleaned and shaped canal space is necessary. The chances of coronal leakage and bacterial recontamination are lowered by proper obturation. It also seals the apex from periapical tissue fluids and the remaining irritants are buried within the canal [14] .

Returning the involved tooth to a healthy and functional condition without surgical involvement, should be the ultimate goal in endodontic treatments [15]. Primarily all inflammatory periapical lesions should be treated with conservative nonsurgical procedures (*e.g.* orthograde root canal therapy) [16]. Only after failing of nonsurgical techniques, surgical intervention is suggested [17]. Moreover, surgery has many disadvantages, which limits its use in the treatment of periapical lesions [18, 19]. Endodontic treatment of teeth with periapical lesions, have been reported to have a success rate of 85% [20, 21]. A 94.4% incidence of complete and partial healing of periapical lesions after nonsurgical endodontic therapy has also been stated [22] .

It is important to note that only through histopathological examination one can make the absolute diagnosis of the nature of the periapical lesion. However, a primary clinical diagnosis of a radicular cyst can be approximately made based on the following facts: If the periapical lesion is a cyst, it is associated with one or more nonvital teeth, the lesion size is usually greater than 200 mm^2^, the lesion is described radiographically as a circumscribed, well-defined radiolucent area with a thin radiopaque lining and finally, it produces a pale, brownish, yellow-colored fluid upon aspiration or when drainage is accomplished throughout the accessed root canal system [23].

**Figure 1:**
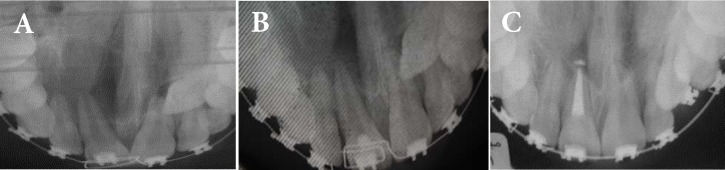
*A)* First visit occlusal view; *B)* After 6-month placement of calcium hydroxide; *C)* After 20 months of calcium hydroxide placement

## Case Report

A 17-year-old male with no notable medical history, good oral hygiene and history of trauma, was referred to the Endodontic Department of Islamic Azad University, with his chief complaint being pain and swelling of the anterior part of upper jaw. The patient was under orthodontic treatment although the orthodontic forces were inactive at that time. According to the patient, nature of the pain was dull, constant and diffused with moderate severity which was spontaneous and aggravated by mastication. 

Extra-oral examination revealed no facial swelling nor palpable lymph nodes. Intra-oral examination showed no injury to soft tissues, no bleeding, intact clinical crown and buccal and palatal fluctuant swelling. Cold, heat and electrical pulp tests (EPT) (Analytic Technology, Redmond, WA, USA) were done, which elicited negative responses. 

Findings from periapical and occlusal radiographies and cone-beam computed tomography (CBCT) showed an extensive periapical radiolucency around the apex of the maxillary right central incisor, pulp canal space was wide and the tooth had an intact crown (Figure 1). The clinical and radiographic findings were suggestive of periapical pathology in relation to maxillary right central and lateral incisors. According to the test results this differential diagnosis was suggested: radicular cyst and periapical granuloma. Moreover, the primary treatment plan was accordingly suggested to be nonsurgical root canal therapy, long-term calcium hydroxide therapy and avoiding orthodontic forces during treatment of the lesion.

**Figure 2:**
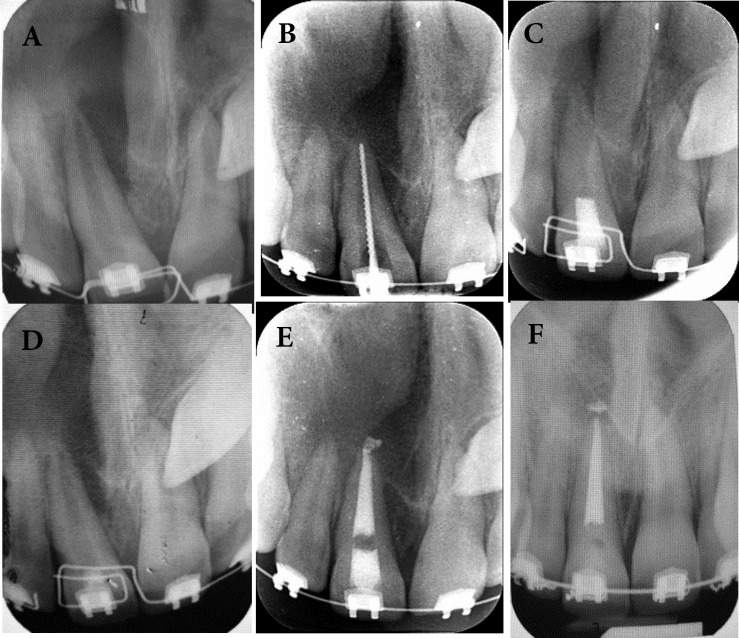
*A)* Pretreatment periapical view; *B)* Working radiography; *C)* Application of calcium hydroxide; *D)* After 6 months of calcium hydroxide therapy; *E)* Post treatment radiography; *F)* Follow-up radiography after 20 months

After administrating local anesthesia (infiltration of 2% Lidocain with 1:80000 epinephrine, Darou Pakhsh, Iran) and rubber dam isolation, the endodontic access cavity was prepared. After radiographic determination of the working length, cleaning and shaping of the canal was done with rotary instruments (step-down technique using RaCe rotary files, FKG Dentaire, La-Chaux-de Fonds, Switzerland) and irrigation with 5.25 % NaOCl (Figure 2A and B). A creamy mixture of calcium hydroxide (Golchadent, Tehran, Iran) was placed in the canal with lentulo spiral (Dentsply Maillefer, Ballaigues, Switzerland) for two weeks and the access cavity was temporarily restored. Before patient dismissal, 400 mg Ibuprofen three times daily was prescribed for two days. 

After two weeks, no signs and symptoms were present and the intra-canal medication was replaced with a thick mixture of calcium hydroxide (Figure 2C).

The third visit was after 6 months and periapical, occlusal and CBCT images were ordered. These radiographies showed significant shrinkage in the lesion size (Figure 2D). However, bone-healing process was detected in periapical radiography and bone formation was confirmed with CBCT. At this point the canal was re-accessed and working length was determined radiographically and by using Root-ZX electronic apex locator (J. Morita USA, Inc., Irvine, CA, USA). Then the preparation was completed with step-down technique with the master apical file (MAF) set at #80. Then the canal was obturated using lateral condensation of gutta-percha points (Dentsply Maillefer, Ballaigues, Switzerland) and AH-26 as sealer (Dentsply, De Trey, Konstanz, Germany) (Figure 2 E). 

**Figure 3:**
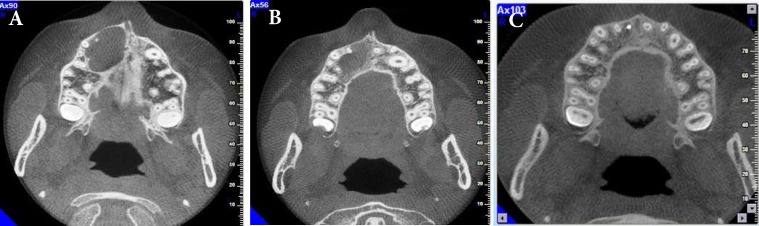
CBCT view; *A)* first visit; *B)* after 6 months, Significant shrinkage in the size of the lesion was observed; *C)* After 20 months of follow-up

After a 20-month follow-up, the patient was asymptomatic and there was no sign of periapical pathology and significant bone formation was seen at the periapical region on periodic follow-up radiographies (Figure 2F). Complete radiographic and clinical healing of the periapical lesion was observed (Figure 3).

## Discussion

Most of the periapical lesions (>90%) can be categorized as dental granulomas, radicular cysts or abscesses [3, 4]. The occurrence of cysts in periapical lesions shows a discrepancy between 6 and 55% [5]. There is clinical indication that as the periapical lesions grow in size, the ratio of radicular cysts rises. However, some large lesions have been shown to be granulomas [24]. The conclusive and definite diagnosis of a cyst can be made only through histopathological findings. In this case, both radicular cyst and periapical granuloma were included in the differential diagnosis list.

The variety of treatment choices for large periapical lesions can be widely made from conventional nonsurgical root canal therapy with calcium hydroxide intracanal medication to various surgical procedures [25]. In this case, the lesion was healed by using nonsurgical endodontic treatment with calcium hydroxide.

A nonsurgical procedure should be done particularly in cases where lesions are close to vital anatomical landmarks. Adequate cleaning, shaping, asepsis and filling of the root canal are the keys to success of the nonsurgical endodontic treatment [25]. In the present study, radiographies showed that the involved teeth had large periradicular lesion with uniformly radiolucency and well-defined margins around the apices. Paduano *et al.* [26] concluded that after endodontic treatment of cyst-like lesions, orthodontic forces can be applied while the lesion is not completely healed. Another case report stated that the periapical lesion of a patient whose orthodontic treatment commenced 2 months before the root canal therapy, completely healed after 2 years of follow-up[27]. However, it has been accounted that if endodontic treatment is necessitated, orthodontic treatment should be deferred until completion of endodontic treatment and clinical and radiographic proof of healing [28]. In this case report, the orthodontic force was inactive before the first visit and after detection of bone-healing in periapical radiography and confirmation of bone formation with CBCT, orthodontic forces were re-activated.

Calcium hydroxide is a widely used intra-canal medicament, because of its high alkalinity [8] and bactericidal effect [29, 30]. It is recommended to place calcium hydroxide in the root canals and into the periradicular tissues while large and chronic periapical lesions are present. It is also believed that it has a direct effect on inflamed tissues and epithelial cystic linings and finally results in periapical healing and bone repair [31]. In this study, calcium hydroxide [10] was placed in the root canal for two weeks and the symptoms were relieved. The lesion showed shrinkage after reapplication of calcium hydroxide for another 6 months; therefor there was no need to use antibiotic paste.

Periapical lesions with endodontic origin, are expected to heal after non-surgical root canal therapy. Earlier, it was thought that 40 to 50% of periapical lesions were cystic. However, it has been discovered that only 15% of these lesions are cysts, while only half of them are true apical cysts [10]. According to Nair [24], there are two types of cysts, a periapical pocket cyst, which is connected with the root canal and a true apical cyst, which is an independent lesion. The true apical cyst needs additional surgical intervention to resolve and a single root canal therapy is not enough.

As in the present case, root canal therapy, provides the highest standards of treatment with consideration of asepsis, adequate cleaning and shaping, irrigation, canal disinfection and careful use of calcium hydroxide, which can cause shrinkage in large periapical lesions. The treatment prognosis of large periradicular lesions is not as good as the small ones. While Strindberg and Sjogren [12] showed no significant differences in healing rate between lesions larger than 5 mm and those smaller than 5 mm, they have also emphasized on the importance of a long-term follow-up for treated teeth with periradicular lesions. In a long time clinical study, 42 non-surgically treated teeth with large cyst-like lesions, were reported that showed complete healing with success rate of 73.8% [32]. In addition, the lesion in the present case was also larger than 5 mm and it was resolved after non-surgical therapy. These results may be attributed to young patient’s rich blood supply, lymphatic drainage and abundant undifferentiated mesenchymal cells of periapical tissues and therefore its good potential for healing [32].

## Conclusion

Successful management of large periapical lesions is achievable with non-surgical root canal therapy and if required, with aspiration, irrigation and antibiotic therapy. Surgical management should be performed if the lesion does not heal.
